# Application of double-pulse laser-induced breakdown spectroscopy (DP-LIBS), Fourier transform infrared micro-spectroscopy and Raman microscopy for the characterization of copper-sulfides

**DOI:** 10.1039/d1ra07189k

**Published:** 2021-12-22

**Authors:** Constantinos Varotsis, Charalampos Tselios, Konstantinos A. Yiannakkos, Charalampos Andreou, Marios Papageorgiou, Antonis Nicolaides

**Affiliations:** Department of Chemical Engineering, Cyprus University of Technology Eirinis 95 Limassol 3041 Cyprus c.varotsis@cut.ac.cy +357 25002802

## Abstract

The combined application of the structure sensitive techniques Fourier transform infrared μ-spectroscopy and Raman microscopy in conjunction with different approaches of laser-induced breakdown spectroscopy (LIBS) including the two-color double pulse (DP-LIBS) have been applied towards the characterization of whole ore copper-sulfide minerals. Discrete information from the surface of the whole ore minerals that lead to the establishment of infrared marker bands and from the surface of bioleached samples that allow the monitoring of jarosite and biofilm formation are provided by FTIR mapping experiments. Raman data can provide information related to the type of the mineral and of the secondary minerals formed on the surface of the ore. Of the four different LIBS approaches applied towards the characterization of the composition of the whole ore minerals, the DP-LIBS shows the highest sensitivity with increasing signals for both the Fe and Cu metals in the whole ore samples.

## Abstract 2

Raman and FTIR microspectroscopies, laser induced breakdown spectroscopy (LIBS) and DP-LIBS have been applied towards our understanding of the characterization of the structure and structure–function relationship in copper-sulfide minerals.

## Graphical abstract

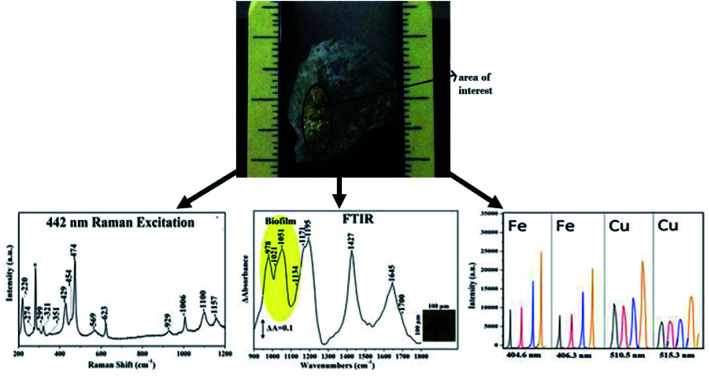

## 1. Introduction

**Fig. 1:**
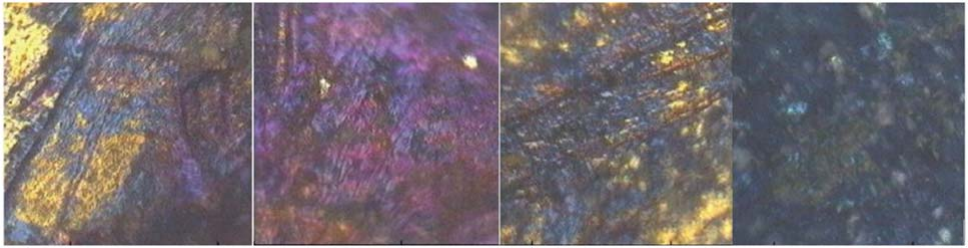
Whole ore copper sulfide minerals with characteristic color layers. Chalcopyrite: golden yellow; idaite: harvest gold; bornite: purple; covellite-chalcocite: blue-navy. The images were taken by a HYPERION 2000 microscope.

The mineralogy, mineral chemistry, geological occurrences, and mineral processing techniques of sulfide minerals have been well investigated and applied because of their economical and environmentally safe significance.^1–49^ There is consensus, that applications of advanced spectroscopic techniques are necessary for the characterization of minerals from whole ores.^3,4,47^ In the field of bio-hydrometallurgy the determination of the chemical and structural properties of minerals prior to microorganism–mineral interactions is the determinant factor towards the successful metal(s) extraction.^3,4^ The technique it is simple and has been successfully applied for Cu extraction from sulfide minerals.^1–4^ Novel techniques have been employed to monitor metal extraction from low-grade sulfide ores and for probing the bacterial activities of acidophilic iron- and sulfur-oxidizing microorganisms towards elimination of Fe^3+^ hydroxysulfates such as jarosite.^13–30^ A great number of studies have agreed in that jarosite precipitation is linked to the passivation of chalcopyrite. There is consensus that the mechanisms involved in biohydrometallurgy require substantial further re-search and thus, new methods towards the fast and accurate identification of the metal content of whole ore minerals and the mechanisms involved in their extraction should be further explored. Laser-induced breakdown spectroscopy (LIBS), Raman and FTIR techniques are attractive tools in the areas of environmental monitoring, geology and in the field of mining. In the mining industry all three techniques have been applied in order to develop sensing technologies that can be used as robust and rugged tools for the positive identification of heavy metals in real-time and because they are cost effective and easier for application (Fig. 1).

The (bio)-chemical oxidation of chalcopyrite [CuFeS_2_], chalcocite [Cu_2_S], idaite [Cu_5_FeS_6_] and bornite [Cu_5_FeS_4_] by microorganisms has been a subject of extensive research for the last twenty years.^1–49^ Oxidation of the most important Cu sulfides (covellite, chalcocite and chalcopyrite) is presented in eqn (1)–(3):

1 CuS(s) + Fe_2_(SO_4_)_3_(aq) → CuSO_4_(aq) + S(s) + 2FeSO_4_(aq)

2 Cu_2_S(s) + Fe_2_(SO_4_)_3_(aq) → 2CuSO_4_(aq) + S(s) + 4FeSO_4_(aq)

3 CuFeS_2_(s) + Fe_2_(SO_4_)_3_(aq) → CuSO_4_(aq) + 2S(s) + 5FeSO_4_(aq)

The passivation of chalcopyrite [CuFeS_2_] is a major problem that reduces the yields from leaching and bioleaching. Passivation involves the formation of a layer of secondary minerals on minerals surface which creates a diffusion barrier to fluxes of re-actants and products. We have presented Raman and FTIR evidence toward identification of secondary minerals formed during the passivation of Cu sulfide minerals in the presence of iron and sulphur-oxidizing bacteria isolated from the mines of HCM.^3,4,47^ The major detectable species in the passivation layer are CuS and elemental sulphur-S^0^. In addition, the surface dynamics of the formation of micro-macro-colonies with variable cell density and the formation of extracellular polymeric substances toward the elucidation of the sulfur bio-oxidation and biofilm format has been reported. The Raman and FTIR data indicated the S_*n*_^2−^/S^0^ consumption modification during biofilm evolution. This way, the role of the cell density in the primary events with the presence of S^0^ was determined and whether the presence of refractory sulfur species on the surface of the mineral affects biofilm formation. The constituents of EPS of the biofilms was also evaluated. Essential elucidation of the mineralogy of [CuFeS_2_] and its transformation is necessary to understand the mechanism of passivation. The main objective of our research has been the characterization of the mineral phases generated in the bioleaching processes and gain insights in transformation processes of these phases, as a support to understand its influence on the passivation of the process.

FTIR spectroscopy is an analytical spectroscopic method for studying subtle changes in secondary structure of proteins as it allows the analysis of chemical bonds.^3,4,47,50–53^ Monitoring the conformations changes of protein structures may originate from interactions with other biomaterials or inorganic matter. Such interactions are of high relevance in various physiological cellular processes such as cellular attachment in sulfide mineral surfaces during bioleaching processing.^47^ Laser Raman micro spectroscopy has been applied as a technique for the characterization of biomaterials and secondary minerals formed during the chalcopyrite leaching.^54–56^ This way, distinct information from a large sample area of a heterogeneous sample is feasible. We have recently applied these microspectroscopic approaches for probing the formation of secondary minerals from the surface of bioleached Cu-containing minerals and also establish the vibrational marker bands for monitoring the formation of different types of jarosite and of EPS.

LIBS has the capability of rapid and *in situ* analysis with no sample preparation required which allows measurements in the laboratory and in the field including studies in geological areas.^57–62^ The specific application of concurrent time resolved LIBS-Raman for monitoring the origin of mining tailings provide a new state-of-the-art approach in the field. This is important at both the level of the industrial heap bioleaching process, and more generally for the scientific community because the time-resolved approach increases the limit of detection. This is a novel approach in heap bioleaching and although it has been used by scientists in other field of biotechnology in the copper bioleaching process has not been applied, yet. LIBS employs a high-power laser beam focused onto a sample to create a plasma. A short duration laser pulse strikes the sample increasing the temperature of the sample causing the matter in the spark to be vaporized, reduced to its atomic species, and then electronically excited. Emission lines at discrete wavelengths that characterize the elements present are then resolved spectrally and temporally with a spectrometer covering part or all of the ultraviolet through near infrared range. Each element emits at different wavelengths and therefore the elements contained within a material can be characterized from the spectral peaks present. The peak intensities provide a quantitative description of the material.

The combination of LIBS-Raman and FTIR mapping techniques provides significant advantage in monitoring chemical-processes and composition because the molecular information is added to the atomic data and a more complete description is available. LIBS is more sensitive in the detection of metal elements than nonmetals. The Raman bands of some materials have well known characterized vibrational bands which can specifically identify the molecular composition or their crystals and crystal form. No reports with respect the applicability of all three techniques in the field of bio-hydrometallurgy are available. In this short review, we have extended our time-resolved vibrational approach of Raman and step-scan FTIR on biomolecules and present new results based on different approaches of LIBS to demonstrate the importance of the techniques towards our understanding the bio-hydrometallurgy dynamics of whole ore sulfide minerals.

## 2. Experimental

### 2.1. Bioleaching experiments

Samples of copper sulfide minerals were collected from the mines of HCM in Skouriotissa, Cyprus, and for the bioleaching experiments the samples were placed in test tubes with the corresponding growth medium and a proper inoculation of cell suspensions of *Acidithiobacillus ferrooxidans* (DSM 14882) and *Erythrobacter Longus* (DSM 6997). The bacteria were purchase from Deutsche Sammlung von Mikroorganismen und Zellkul-turem (DSMZ). The bacteria were cultured in Difco marine broth 2216 medium at 28 °C. Bio-interaction experiments in the tubes were carried out under aseptic conditions in a water bath at 37 °C using recirculating solutions. The test tubes were fitted with rubber stoppers and the suspension (growth medium and cells) was recirculated by a pump from Cole-Parmer through inlet/outlet tubes. The test tubes were placed in a custom-made plexiglass apparatus of 60 positions.^3,4^

### 2.2. FTIR microspectroscopy

**Fig. 2:**
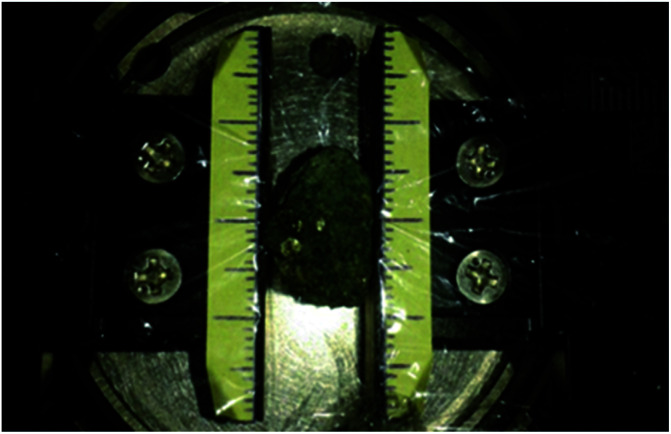
Sample stage used for the experiments of the whole ore samples.

Fourier transform infrared microspectroscopy (μ-FTIR) was applied at defined time intervals to monitor the conformational changes in amide I during the biofilm formation and jarosite formation on the copper sulfide ores. Spectra were collected with a Tensor 27 Fourier transform infrared spectrometer and a coupled HYPE-RION 2000 microscope. The microscope was equipped with a liquid nitrogen cooled HgCdTe (MCT) detector and a motorized *xy* sample stage. Spectra acquisitions were obtained at a resolution of 4 cm^−1^ over the spectral range 7500–600 cm^−1^ using a co-addition of 32 scans and a defined optical field of 100 × 100 μm by glass slide. Spectra were recorded in reflectance mode using a 15× or 36× IR objective and processed through Opus 7.0 (Bruker) and OriginPro 9.0 softwares. Attenuated total reflection was used to obtain infrared spectra of extracellular polymeric substances in film form. Spectra were collected using an ATR-Germanium plate (Pike Technologies) and the FTIR Tensor 27 (Bruker) spectrometer equipped with a deuterated triglyceride sulfate (DTGS) detector. The spectra were collected in the 900–4000 cm^−1^ spectral range with a resolution of 4 cm^−1^ and 100 co-exposures. Prior to each sample measurement, a back-ground spectrum was collected. The OPUS 7/IR (Bruker) software package was used to acquire and process the FTIR spectra. Fig. 2 depicts the sample stage used for the experiments of the whole ore copper sulfide minerals.

### 2.3. Raman microspectroscopy

Raman data were collected by a LabRAM from HORIBA Jobin Yvon equipped with a CCD detector. It is equipped with an Olympus BX41 microscope 50X. The 442 nm excitation laser beam was provided by a Helium–Cadmium laser. The laser power incident on the sample was 20 mW and the accumulation time 15–20 min for each spectrum.

### 2.4. Two color double pulse LIBS

**Fig. 3:**
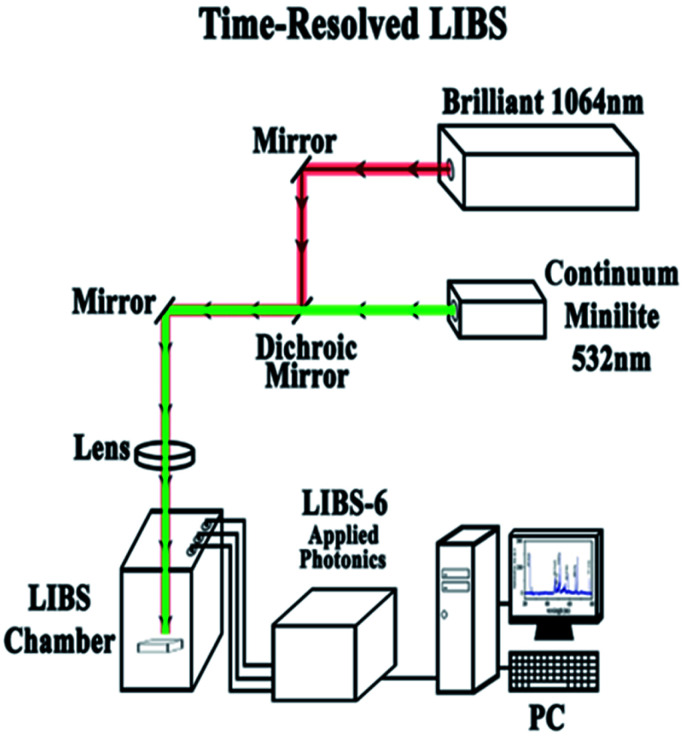
Experimental set up for the two color (1064 and 532 nm) DB-LIBS. The experimental set-up consists of two Nd:YAG lasers Brilliant (Quantel) operating at 10 Hz and Minilite (Con-tinuum) operating at 1–15 HZ and the LIBS-6 from applied photonics. In the time-resolved DP-LIBS a delay generator (Stanford Instruments Model DG535) controls the triggering and timing of the lasers.

Fig. 3 shows the experimental set-up for the time-resolved LIBS that can be used as a single pulse (1064 or 532 nm), dual pulse (collinear 1064 and 532 nm) and in a time-resolved approach. The present study investigates the single and the collinear two-color double-pulse LIBS (DP-LIBS) configuration using 1064 nm from a Quantel Brilliant nanosecond laser (7 ns pulse) operating at 10 Hz or 50 Hz and the 532 nm pulse from a Minilite Continuum laser (7 ns pulse) operating at 1–15 Hz. For dual pulse experiments a delay generator (Stanford Instruments Model DG535) will control the triggering and timing of the lasers. We will utilize the double pulse technique with delay time *t*_d_ = 1.5 ns which is ideal to detect and visualize the distribution of the desired chemical element on the sample surface. The LIBS laser beam that was used for the experiments reported in this paper was the Brilliant from Quantel at a wavelength of 1064 nm. Every spectrum was taken with pulsed energy of 30 mJ at a repetition rate of 10 Hz. The internal delay time was 2.51 μs and the integration time 0.030 ms. In addition, the Minilite laser was used from continuum at a wavelength of 532 nm and at a rep rate of either 1 Hz or 10 Hz and pulsed energy of 8 mJ. A spectrometer from applied photonics was used to analyze plasma emission.

## 3. Results and discussion

We have performed Raman, FTIR and LIBS on different types of whole ore copper sulfide minerals. Raman spectroscopy is a powerful structure sensitive technique for identifying species on the surfaces of the whole copper sulfide minerals. A number of sulphides exist in the Cu/Fe/S system that contain S–S bonds including pyrite, FeS_2_ which displays a S–S stretching vibration at 380 cm^−1^ and is significantly different from that present in the Cu/Fe/S system that contain S–S bonds and has been observed at 464–467 cm^−1^.^34–47^ Thus, CuS can be distinguished from other phases that contain S–S bonds, and the position of the S–S stretch provides information on the Fe content of the phase. Elemental sulphur and polysulfides also contain S–S linkages and display sharp S–S stretch bands in the Raman spectra at 470 cm^−1^. This band overlaps with that from CuS and bornite [Cu_5_FeS_4_] bornite when both species are present on the same region of the surface. Obviously, covellite and bornite cannot be distinguished from sulphur from this band alone. Sulphur also displays an equally strong band from S–S–S chain bending at 215 cm^−1^ which is not found in covellite and the ratio of the intensities of the two bands can potentially be used to distinguish the two phases.

**Fig. 4:**
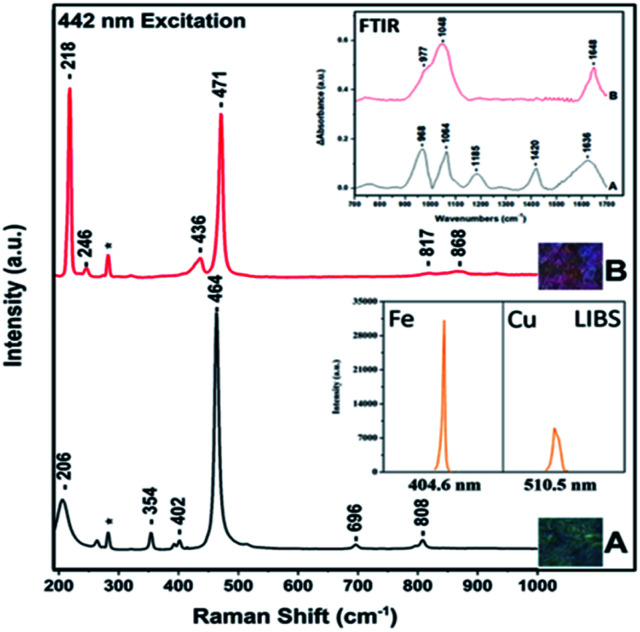
442 nm Raman spectra of quartz (spectrum A) and bornite Cu_5_FeS_4_ (spectrum B). Ex-perimental conditions are the same as previously reported (3,4). The inset shows the FTIR spectra of (A) chalcopyrite (B) idaite.

The Raman spectrum of chalcopyrite is characterized by a weak band at 293 cm^−1^ which has been assigned to the symmetric anion A1 mode.^21,22^ The *v*(Cu–S) stretching vibration in the Cu/Fe/S systems, bornite, chalcocite and covellite is located in the 470–474 cm^−1^. Therefore, it is difficult to distinguish bornite, chalcocite and covellite based on the *v*(Cu–S). However, there are easily distinguishable by colour. The *v*(Cu–S) of Cu_2_S which belongs to a Cu/S system is observed at 470 cm^−1^ providing new information on the iron content in the Cu/Fe/S system. The *v*(Cu–S) of chalcocite was recently reported for the first time.^4^ It was previously reported that CuS_2_ has no Raman peaks between 200 and 500 cm^−1^ and thus its formation was suggested without providing evidence for its formation.^21,22^ The Raman peak at 474 cm^−1^ is the *v*(Cu–S), in agreement with previous results.^21,22^ The Raman spectra including the images can provide the necessary information for characterization of surface species/phases and spatial variations in composition of the bioleaching of chalcopyrite. Furthermore, information regarding the formation of the passivation layers in all of the above mentioned Cu/Fe/S and Cu/S systems is feasible. Fig. 4 spectra A–B depicts the 442 nm excitation Raman spectra of quartz (spectrum A), and bornite (spectrum) and in the inset the FTIR spectra of chalcopyrite (spectrum A) and idaite (spectrum B). The image of the sample (spectrum A) has some colour-characteristics of that of either covellite and or chalcocite. However, the Raman spectrum shows an intense band at 464 cm^−1^ (A1 mode) and a moderate at 206 cm^−1^ which both have been reported to originate from quartz. Spectrum B is similar to that we have reported.^3,4^ As observed in the inset spectrum A, which corresponds to chalcopyrite, the stronger signals could correspond to ferric sulfate at 1064 cm^−1^, and hydrated ferrous sulfate and copper sulfate at 968 cm^−1^, ferrous sulfate and hydrated ferric sulfate (1185 cm^−1^). The broad band at 1636 cm^−1^ is due to H–O–H. Spectrum B which corresponds to idaite has characteristic bands of 977 cm^−1^ due to hydrated ferrous sulfate and copper sulfate at 968 cm^−1^ and at 1048 cm^−1^ due to ferric sulfates. For the FeS_2_ samples, the main peaks correspond to hydroxyl ferric sulfate (1230 cm^−1^), ferric sulfate (1176 cm^−1^), ferric sulfate cluster (1140 cm^−1^), ferrous sulfate (1089 cm^−1^) and hydrated ferric sulfate (1020 cm^−1^). Non-hydrated ferric sulfates possess strong vibrations in the 1200 cm^−1^ range, and, as observed in Fig. 6, there is almost no existence of these vibrations in the case of idaite but in the case of chalcopyrite there is a band at 1185 cm^−1^.

**Fig. 5:**
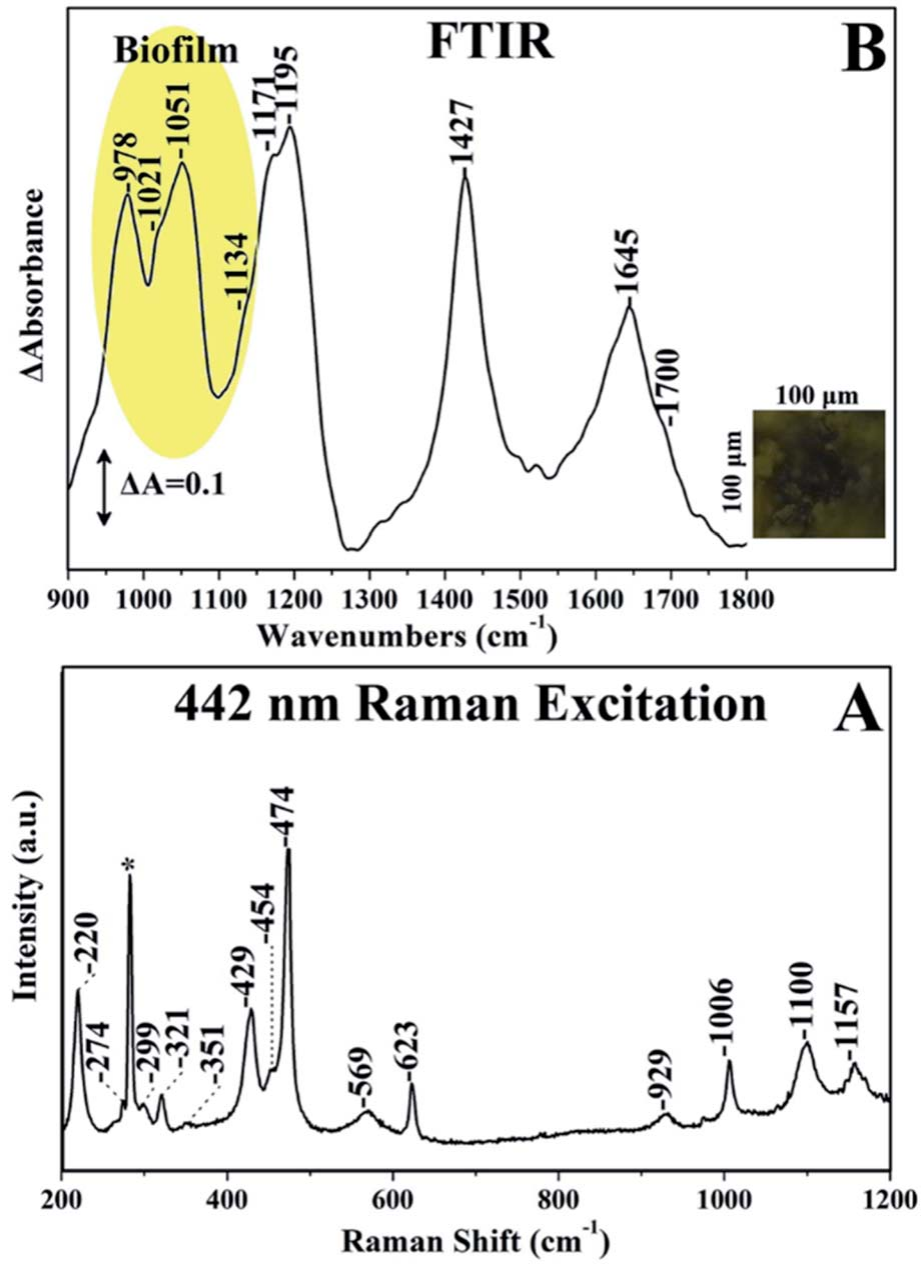
Panel A: 442 nm Raman excitation spectra of bioleached bornite by A. The laser power incident on the sample was 20 mW and the accumulation time was 15 minutes. Panel B: collective image of surface of bioleached bornite [Cu_5_FeS_4_] by *Acidithiobacillus ferrooxidans* and the FTIR spectra collected form the surface of the mineral. The area of infrared fingerprint is 0.01 mm^2^ with spectra resolution 4 cm^−1^.^47^

Fig. 5 shows the Raman and FTIR spectrum of bornite [Cu_5_FeS_4_] in a period of ten months of bioleaching by *A. Ferrooxidans*. In a recent paper the detailed analysis and assignment of all the vibrations present in the Raman and FTIR data were reported.^3,4,47^ The K^+^ and NH_4_^+^ vibrations of jarosite is the *ν*_3_(SO_4_^2−^) vibration which is located observed at 1100 cm^−1^ in the spectra of K^+^-jarosite and at 1091 cm^−1^ in the spectrum of NH_4_^+^-jarosite^3^ and the band at 474 cm^−1^ was assigned to *ν*(Cu–S) of covellite.^47^ Elemental sulphur and polysulfides contain S–S linkages and display the *ν*(S–S) and *δ*(S–S–S) which are characterized by equally strong intensities at 470 at 215 cm^−1^, respectively. The intensity of the *ν*(S–S)/*δ*(S–S–S) ratio at 470/215 = 1 has been applied used to distinguish the elemental sulphur from the other S–S and Cu–S bond containing species.^3,4^ In addition, all bands attributed to K^+^-jarosite including the *ν*(Fe–O) at 429 cm^−1^ are present.^47^

In the FTIR spectra shown in Fig. 5B all the characteristic bands were assigned to amide I (1645 cm^−1^), to biofilm formation (978–1134 cm^−1^) and near 1700 cm^−1^ to the *v*(C=O) vibration of the O-acetyl ester bond of free EPS. The bands at 978, 1021 and 1051 cm^−1^ are due to carbohydrates and at 1134 cm^−1^ due to P=O and the band at 1171 cm^−1^ was attributed to Fe–O–P bonds.^47^

Potassium Jarosite [KFe_3_(SO_4_)_2_(OH)_6_] is common in acidic and sulfate-rich environments. Jarosite typically is formed in ferric rich, acidic (pH < 3) oxic environments and readily breaks down when removed from its stability region by presumably converting to iron(iii) oxide or oxyhydroxide phases. K^+^-jarosite converts to goethite through the following reaction^43^

4 KFe_3_(SO_4_)_2_(OH)_6_(s) ↔ 3FeO(OH)Geothite + K^+^(aq) + 2SO_4_^2−^(aq) + 3H^+^(aq)

However, the mechanisms of this reaction, and the specific products formed have not been studied in detail. This knowledge is of profound importance in predicting geochemical reactions in metallurgical environments. Monitoring the fate of K, SO_4_^2−^ and Fe during K-jarosite dissolution will aid in describing the geochemical cycling of these element in ARD/AMD systems. Jarosite which is formed subsequently to the formation of goethite has a low solubility and high stability (*K*_sp_ = 10^−11^ and *G*_0_^*f*^ = −3309.8 kJ mol^−1^). The formation of jarosite is also related to the redox potential of the solution. Redox potential higher than 400 mV favour Fe^3+^ precipitation. It has been demonstrated that *Acidithiobacillus ferrooxidans* is forming jarosite group minerals de-pending on the temperature, pH, aging time and dissolved oxygen (OD) levels.^10,18^ From the dissolution rates we can determine if K-jarosite can survive as long as the NH_4_^+^-jarosite. Jarosite in biological environments is formed from schwertmannite Fe_8_O_8_(OH)_6_SO_4_ which is substituted with NH_4_^+^/H_3_O^+^ when the temperature is increased from 36 to 45 °C. The required cations can be acquired from dissolution of minerals and from acid-neutralizing additives.

Electron scanning microscopy (SEM) images of unleached and leached chalcopyrite have been reported but it is difficult to differentiate K^+^-from NH_4_^+^-jarosite because jarosite morphology depends on the experimental conditions and the shape which is round for K^+^-jarosite and cubic for the NH_4_^+^-jarosite and size cannot be used as a tool. On the same line are the X-ray diffraction patterns which are difficult to differentiate on the basis of *d*-spacing. The formation of K^+^ and NH_4_^+^-jarosite has been observed in 35–100 mm size grains of high-grade chalcopyrite after bioleaching in laboratory studies but never observed in natural ore samples under experimental conditions that mimic chalcopyrite heap bioleaching processes at 35 °C. In addition, the communities of microorganisms (biofilms) which are embedded in a hydrogen-like matrix formed by EPS on chalcopyrite surfaces during the bioleaching process contributing in the biosynthesis of jarosite have not been reported yet. An overall equation for the dihydroxylation, dehydration and deammoniation of NH_4_^+^-jarosite in the 300–400 °C range is given by

5 2NH_4_Fe_3_(SO_4_)_2_(OH)_6_ → 7H_2_O + 2NH_3_ + 2Fe_3_O_2.5_(SO_4_)_2_

**Fig. 6:**
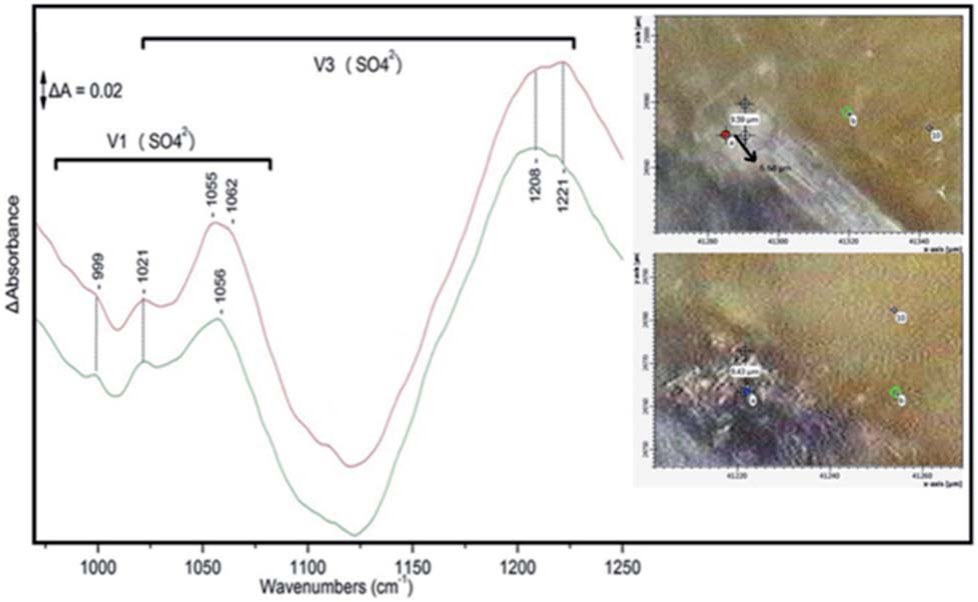
The FTIR and the 100 × 100 mm FTIR spectra of chalcopyrite which showed changes of the ore over a six-month bioleached time period have been reported by our group.

Fig. 6 shows the FTIR spectra and images of a bioleached area of covellite with *A. ferroxidans* in a two month period. The bottom spectrum is that of the first month with characteristic bands due to *v*_1_ and *v*_3_ of (SO_4_). In a time-period of two months the top spectrum shows variation in the frequencies of *v*_1_ and *v*_3_ demonstrating the real time changes that take time on the surface of the bioleached ore. Comparison of the images also suggest the formation of biofilm on the surface. This way we can monitor the kinetics of biofilm formation and gain knowledge of the dynamics of the surface proper-ties in the presence of microorganisms and also the time period the microorganisms are active on the surface of the ore. A detailed description of the kinetics involved in the formation of biofilm and jarosite is described in references.

Although sulfide minerals are important sources of metals, they are potential sources of pollution. The release of sulfur through the weathering of sulfides in natural rocks or in mine wastes generates sulfuric acid, resulting in acid rock drainage or acid mine drainage (AMD). Sulfurous fumes are produced by smelting sulfide ores which react to form acid rain. Metal sulfides are very important in Marine geosciences. Therefore, it is intriguing to gain knowledge on the interactions of the most abundant sulfide mineral, FeS_2_ with anaerobic marine phototrophic microorganisms. Several theories have been developed following the discoveries of ocean-floor hydrothermal systems generating large volumes of sulfide minerals and associated with novel life forms and ecosystems.

**Fig. 7:**
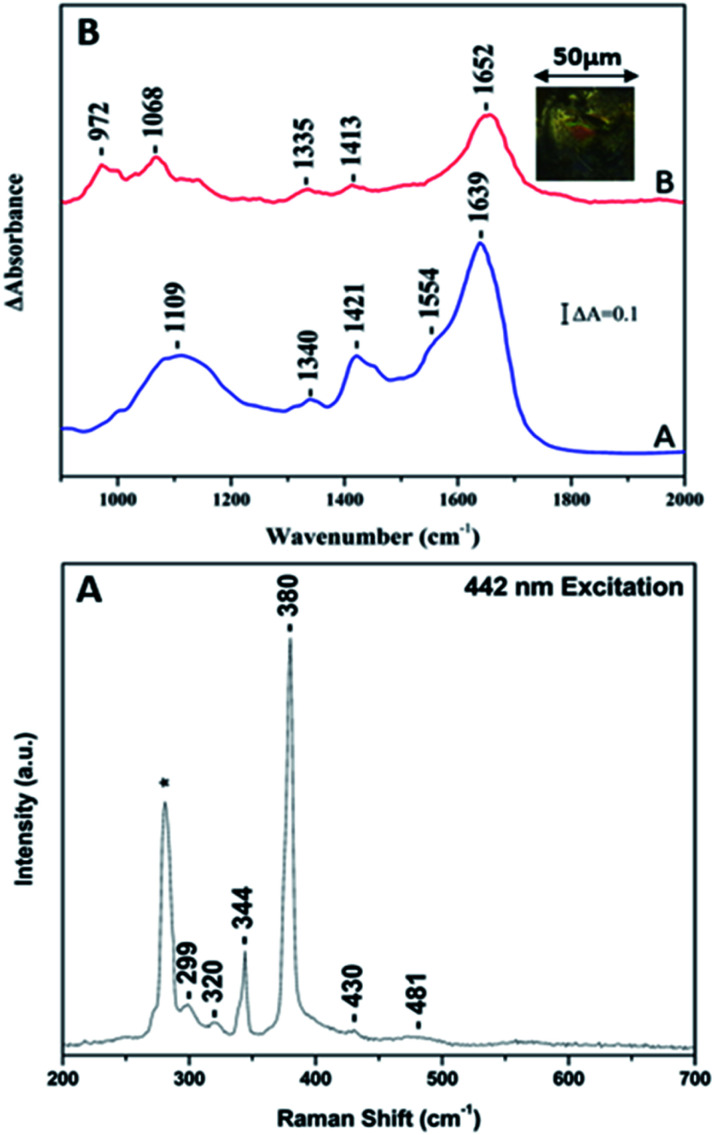
Panel A: 442 nm Raman excitation spectra of the FeS_2_ in the presence of *Erythrobacter Longus*. The laser power incident on the sample was 20 mW and the accumulation time was 15 minutes. Panel B: collective image of *Erythrobacter Longus* (spectrum A) and of the surface of the FeS_2_-*E. Longus* complex. The area of infrared fingerprint is 0.005 mm^2^ with spectra resolution 4 cm^−1^.

Fig. 7 shows the Raman and FTIR spectrum of whole ore mineral containing FeS_2_ in a period of one week of interaction(s) with *Erythrobacter Longus*. In panel A, the in-tense Raman bands at 344 and 380 and 430 cm^−1^ originate from FeS_2_. More specifically, the 380 cm^−1^ band originates from the Ag in plane stretch S–S, the 344 cm^−1^ from the S–S out of phase *E*_g_ mode and the 430 which has been assigned to *T*_g_ coupled libration and stretch mode. The full agreement of the Raman bands of the whole ore FeS_2_ with those reports from pure FeS_2_ samples demonstrates the reliability of the Raman spectroscopy in the characterization of whole ore minerals.

In Fig. 7, panel B, The FTIR spectrum of *E. Longus* (spectrum A) and the 50 × 50 μm FTIR imaging spectrum of *E. Longus* over the one week period of interaction with the FeS_2_ surface (spectrum B). The FTIR spectrum of *E. Longus* is characteristic of the bands at 1639 and 1554 cm^−1^ which are assign to the amide I and amide II, respectively. There are additional bands in the 1420 and 1100 cm^−1^ range due to NH_4_^+^ and carbohydrates, respectively. The well separated bands at 978, 1021 and 1051 cm^−1^ are due to carbohydrates and at 1134 cm^−1^ due to P=O. In spectrum B the appearance of band in the 970–1070 cm^−1^ indicates the presence of EPS biofilm formation. In addition, the am-ide II vibration has lost its intensity and the amide I vibration is up-shifted by 13 cm^−1^ indicating that the proteins of the EPS–FeS_2_ complex are involved in the EPS adsorption on FeS_2_.

LIBS is a relatively simple spectroscopic technique with various advantages that has been applied in a number of experiments and provide sustained, *in situ*, elemental detection in real time.^57–62^ In addition of having the ability to rapidly analyse the elemental composition of solids, liquids, and gases with little or no sample preparation, LIBS is one of the few techniques capable of non-contact and remote elemental analysis, making it particularly useful for analyses in extreme and hostile environments. The application of single pulse LIBS and development of dual-pulse LIBS (DP-LIBS) and resonance-enhanced LIBS for decreasing the limit of detection and im-proving reproducibility of LIBS in agricultural soil and nutrients has been applied towards establishing new methods for improving the detection limit.^61,62^

**Fig. 8:**
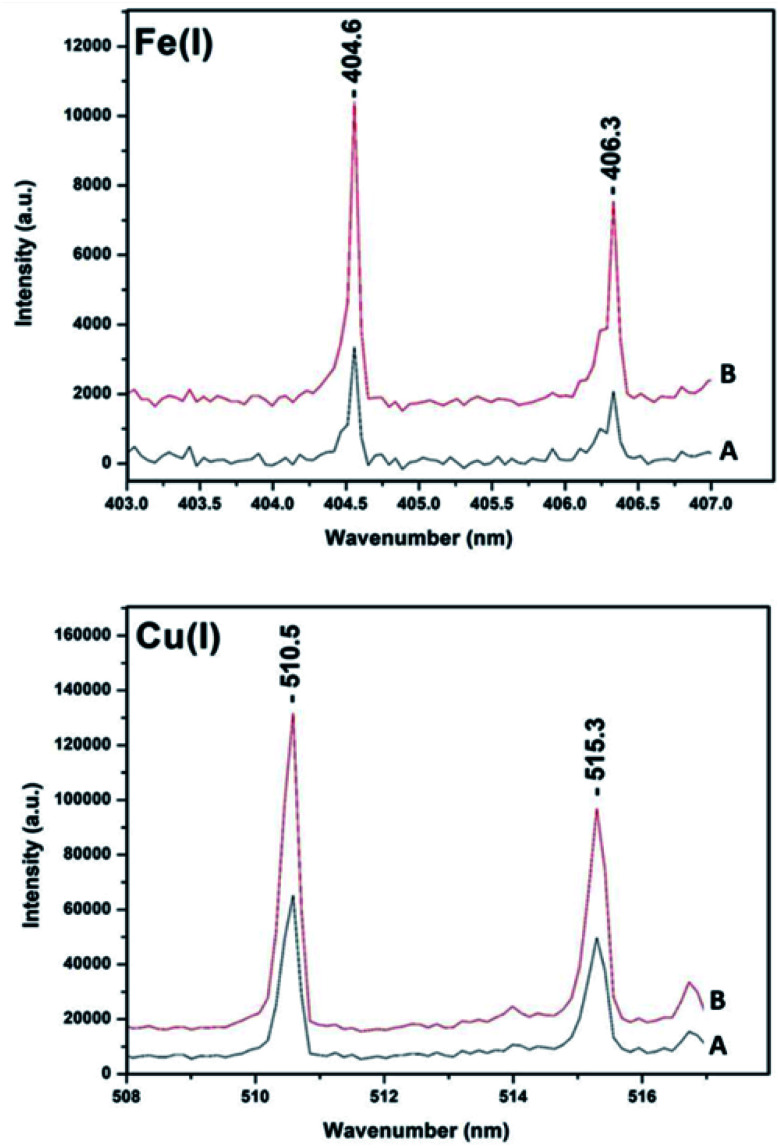
Comparison of single pulse (spectrum A, 1064 nm) with the collinear DP-LIBS (spectrum B, 532–1064 nm) signal intensity for natural Cu and Fe species. The Cu I lines are located at 510.5 nm and 521.8 nm and those of Fe(i) at 404.6 nm and 406.3 nm.

Fig. 8 shows large enhancement in LIBS signals for 532–1064 nm combination in collinear arrangement. For all the comparisons the 1064 nm pulse energy and the in-ternal gate delay kept constant. The spot size for the 1064 nm was approximately 120 micrometers while for the 532 nm was 0.5 mm. The time-delay between the two laser pulses was approximately *t*_d_ = 1–2 ns. It can be clearly seen that there is enhancement in signal intensity for the collinear DP-LIBS. For all measurements a fixed gate delay and *t*_d_ was used. It remains to be established whether the intensity signals depend on the gate delay and *t*_d_. Several mechanisms have been reported suggesting that the increase is due to either the increase in mass ablation or reheating of the plasma plume. The mass ablation rate depends on the wavelength because the shorter wavelength provides stronger laser-target coupling due to higher critical density (*n*_c_*α*1/*λ*^−2^).

**Fig. 9:**
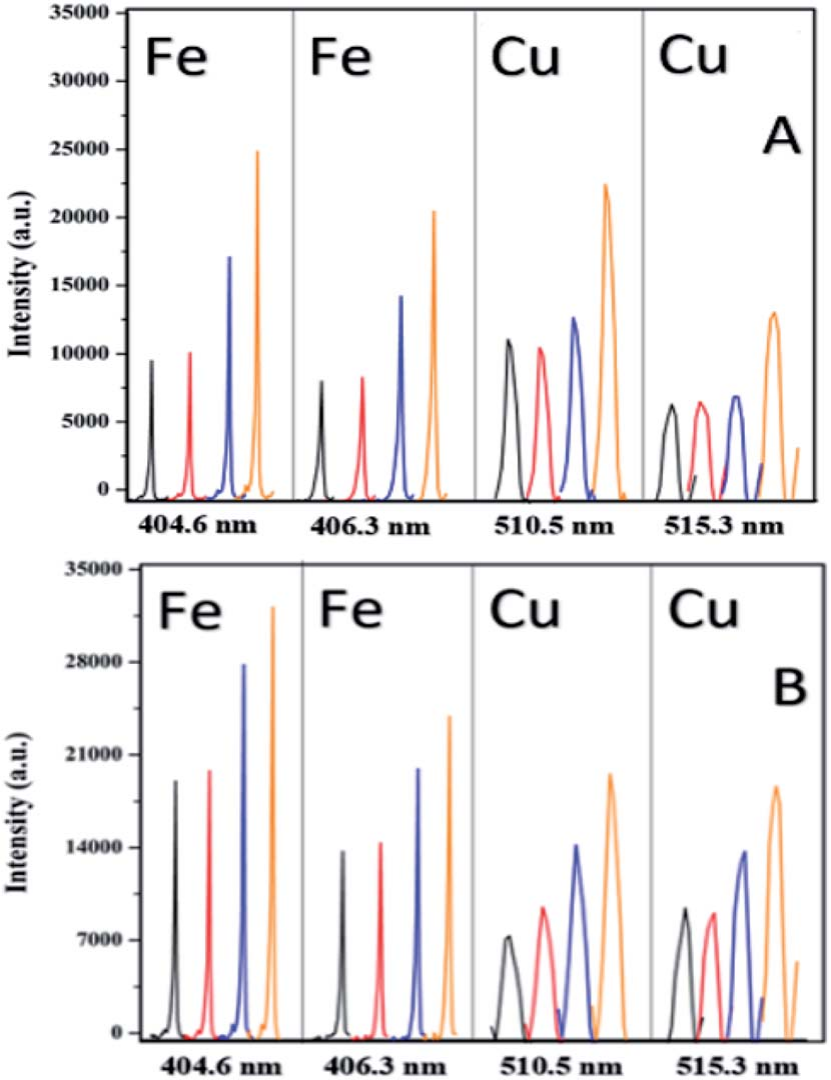
Single 1064 nm LIBS (black), 532 nm prepulse to 1064 nm LIBS (red), 1064 nm prepulse to 1064 nm LIBS (blue) and 532–1064 nm with *t*_d_ = 1–2 ns an internal pulse delay 2.41 microseconds DP-LIBS (orange) of chalcopyrite and idaite. The samples are the same used in the Raman and FTIR experiments.

We have extended the single and DP-LIBS approaches and applied two additional LIBS methods using the same experimental set-up. In the third approach we have used the 532 nm pulse as a pre-pulse to generate the plasma prior to the use of the 1064 nm pulse for recording the LIBS and in the fourth approach in a similar configuration but using the 1064 nm for the pre-pulse and subsequently the 1064 nm for recording the LIBS. We have applied the four-way approach to Cu/Fe/S containing ores including chalcopyrite and idaite whole ore samples. There is a sequential signal increase in going from the single pulse (1064 nm, black line) to prepulse-LIBS (532–1064 nm, red line) to prepulse-LIBS(1064–1064 nm, blue line) and finally to collinear DP (532–1064 nm, orange line) (Fig. 9).

It is shown that this approach can achieve typical double pulse improvement in the analytical performances for elemental analysis chalcopyrite containing ores. The results show a significant increase of the intensity and repeatability of the emission signals in the double pulse configuration of both Fe and Cu contents in the chalcopyrite ore. The improvement resulting from the use of DP-LIBS was about 100% when com-pared to SP-LIBS in all cases. These results are useful in the context of studies investigating how the μ-LIBS will assist in the development of portable LIBS systems and improve the utility of this spectroscopy technique for field applications.^63–68^

## 4. Conclusions

The combination of LIBS with μ-FTIR and μ-Raman provides a unique opportunity to probe the content of whole ore minerals, to characterize the minerals and their interactions with microorganisms. The FTIR mapping experiments provide information related to the adsorption induced variation on the bioleached ores. The present work extends our previous investigations of the Cu/Fe/S containing minerals and indicate that similar approaches are feasible for any type of metal containing minerals. It should be noted that the application of single LIBS it is much easier than the DP-LIBS and it is not absolutely necessary to double the signal intensity to identify the minerals.

Time-resolved Raman and TR- FTIR spectroscopies have been applied extensively in the past for the investigation of biochemical mechanisms of nitric oxide and dioxygen respiration by cytochrome oxidases, nitric oxide reductases and the interactions of microorganisms with surfaces and metals in our laboratory.^22–35,69–71^ The present work extends our previous investigation and the detailed analysis of the data obtained with all three techniques demonstrates the necessity for their concurrent ap-plication towards the elucidation of complex systems such whole ore minerals.

## Conflicts of interest

There are no conflicts to declare.

## Supplementary Material
